# Supplementary material for: Impact of Low Muscle Mass on Hepatocellular Carcinoma Patients Undergoing Transcatheter Liver-Directed Therapies: Systematic Review & Meta-Analysis
Source: Cancers (Basel). 2024 Jan 11;16(2):319. doi: 10.3390/cancers16020319 (PMC10813967; doi:10.3390/cancers16020319)

Supplemental Materials

Table S1 - PRISMA checklist.

Table S2 - Search strategy.

Table S3 - Excluded studies and reasons.

Table S4- Quality assessment of the included cohort studies using Newcastle-Ottawa Scale.

Table S5 - Subgroup analysis of the prevalence of LSMM in HCC patients treated with transcatheter liver-directed intra-arterial therapies.

Table S6 - Subgroup analysis of HR and 95% confidence interval of overall survival in HCC patients treated with transcatheter liver-directed intra-arterial therapies with and without LSMM.

Figure S1. Publication bias.

Figure S2. Sensitivity analysis.

Table S1-prisma checklist

Section/topic	#	Checklist item	Reported on page #
**TITLE**			
Title	1	Identify the report as a systematic review, meta-analysis, or both.	P1
**ABSTRACT**			
Structured summary	2	Provide a structured summary including, as applicable: background; objectives; data sources; study eligibility criteria, participants, and interventions; study appraisal and synthesis methods; results; limitations; conclusions and implications of key findings; systematic review registration number.	P3
**INTRODUCTION**			
Rationale	3	Describe the rationale for the review in the context of what is already known.	P6
Objectives	4	Provide an explicit statement of questions being addressed with reference to participants, interventions, comparisons, outcomes, and study design (PICOS).	P7
**METHODS**			
Protocol and registration	5	Indicate if a review protocol exists, if and where it can be accessed (e.g., Web address), and, if available, provide registration information including registration number.	P8
Eligibility criteria	6	Specify study characteristics (e.g., PICOS, length of follow-up) and report characteristics (e.g., years considered, language, publication status) used as criteria for eligibility, giving rationale.	P8
Information sources	7	Describe all information sources (e.g., databases with dates of coverage, contact with study authors to identify additional studies) in the search and date last searched.	P8

Search	8	Present full electronic search strategy for at least one database, including any limits used, such that it could be repeated.	P8
Study selection	9	State the process for selecting studies (i.e., screening, eligibility, included in systematic review, and, if applicable, included in the meta-analysis).	P9
Data collection process	10	Describe method of data extraction from reports (e.g., piloted forms, independently, in duplicate) and any processes for obtaining and confirming data from investigators.	P9
Data items	11	List and define all variables for which data were sought (e.g., PICOS, funding sources) and any assumptions and simplifications made.	P9
Risk of bias in individual studies	12	Describe methods used for assessing risk of bias of individual studies (including specification of whether this was done at the study or outcome level), and how this information is to be used in any data synthesis.	P9
Summary measures	13	State the principal summary measures (e.g., risk ratio, difference in means).	P9
Synthesis of results	14	Describe the methods of handling data and combining results of studies, if done, including measures of consistency (e.g., I^2) for each meta-analysis.	P9
Risk of bias across studies	15	Specify any assessment of risk of bias that may affect the cumulative evidence (e.g., publication bias, selective reporting within studies).	P10
Additional analyses	16	Describe methods of additional analyses (e.g., sensitivity or subgroup analyses, meta-regression), if done, indicating which were pre-specified.	P10
**RESULTS**			
Study selection	17	Give numbers of studies screened, assessed for eligibility, and included in the review, with reasons for exclusions at each stage, ideally with a flow diagram.	Figure 1
Study characteristics	18	For each study, present characteristics for which data were extracted (e.g., study size, PICOS, follow-up period) and provide the citations.	Table 1

Risk of bias within studies	19	Present data on risk of bias of each study and, if available, any outcome level assessment (see item 12).	Table 2
Results of individual studies	20	For all outcomes considered (benefits or harms), present, for each study: (a) simple summary data for each intervention group (b) effect estimates and confidence intervals, ideally with a forest plot.	Table 1
Synthesis of results	21	Present results of each meta-analysis done, including confidence intervals and measures of consistency.	P12-13
Risk of bias across studies	22	Present results of any assessment of risk of bias across studies (see Item 15).	P 13
Additional analysis	23	Give results of additional analyses, if done (e.g., sensitivity or subgroup analyses, meta-regression [see Item 16]).	Figure s1 s2
**DISCUSSION**			
Summary of evidence	24	Summarize the main findings including the strength of evidence for each main outcome; consider their relevance to key groups (e.g., healthcare providers, users, and policy makers).	P14-17
Limitations	25	Discuss limitations at study and outcome level (e.g., risk of bias), and at review-level (e.g., incomplete retrieval of identified research, reporting bias).	P18
Conclusions	26	Provide a general interpretation of the results in the context of other evidence, and implications for future research.	P19
**FUNDING**			
Funding	27	Describe sources of funding for the systematic review and other support (e.g., supply of data); role of funders for the systematic review.	P4

Table S2 search strategy

Database	Keyword	Date	Results
PubMed	(liver cancer) AND ((((sarcopenia) OR (((muscle mass) AND (hand strength)) AND (walking speed))) OR ((muscle mass) AND (hand strength))) OR ((muscle mass) AND (walking speed)))	October 27,2023	388
Embase	((((("muscle mass") AND (hand strength)) AND (walking speed)) OR (("muscle mass") AND (hand strength))) OR (("muscle mass") AND (walking speed))) OR (sarcopenia)) AND (liver cancer)	October 27,2023	743

Table S3 Excluded studies and reasons

Reason:

1. Studies primarily focusing on hepatic tumors other than HCC;
2. Populations unrelated to LSMM or muscle mass;
3. Populations receiving therapy other than transcatheter liver-directed intra-arterial therapies;

Citation	Reason
Associations of low handgrip strength with cancer mortality: a multicentre observational study	1
Montano-Loza, Aldo MD1; Meza-Junco, Judith MD2; Prado, Carla PhD2; Lieffers, Jessica MSc, RD2; Baracos, Vickie PhD2; Bain, Vincent MD1; Sawyer, Michael MD2. Frequency and Clinical Impact of Sarcopenia in Cirrhotic Patients with and without Hepatocellular Carcinoma: 317. American Journal of Gastroenterology 106():p S125-S126, October 2011.	1
IMPACT OF PRE-TRANSPLANT SARCOPENIA ON SHORT TO MEDIUM TERM OUTCOME IN LIVER TRANSPLANT RECIPIENTS WITH AND WITHOUT HEPATOCELLULAR CARCINOMA.D’ D’ Arcangelo et al. Hepatoma Res 2021;7:4 DOI: 10.20517/2394-5079.2020.109.	1
Dodson RM, Firoozmand A, Hyder O, Tacher V, Cosgrove DP, Bhagat N, Herman JM, Wolfgang CL, Geschwind JF, Kamel IR, Pawlik TM. Impact of sarcopenia on outcomes following intra-arterial therapy of hepatic malignancies. J Gastrointest Surg. 2013 Dec;17(12):2123-32. doi: 10.1007/s11605-013-2348-5. Epub 2013 Sep 25. PMID: 24065364; PMCID: PMC3982291.	1
Kamo N, Kaido T, Hamaguchi Y, Okumura S, Kobayashi A, Shirai H, Yao S, Yagi S, Uemoto S. Impact of sarcopenic obesity on outcomes in patients undergoing living donor liver transplantation. Clin Nutr. 2019 Oct;38(5):2202-2209. doi: 10.1016/j.clnu.2018.09.019. Epub 2018 Sep 28. PMID: 30482562.	1
Montano-Loza, Aldo MD, MSc1; Meza-Junco, Judith MD2; Tandon, Puneeta MD1; Baracos, Vickie PhD2; Bain, Vince MD1; Ma, Mang MD1; Beaumont, Crystal BSc2; Esfandiari, Nina BSc2; Sawyer, Michael MD2; Kneteman, Norman MD, MSc, FRCSC3. Muscle Wasting Is Not Associated With Higher Mortality After Liver Transplantation: 384. American Journal of Gastroenterology 107():p S162, October 2012.	1
Valero V 3rd, Amini N, Spolverato G, Weiss MJ, Hirose K, Dagher NN, Wolfgang CL, Cameron AA, Philosophe B, Kamel IR, Pawlik TM. Sarcopenia adversely impacts postoperative complications following resection or transplantation in patients with primary liver tumors. J Gastrointest Surg. 2015 Feb;19(2):272-81. doi: 10.1007/s11605-014-2680-4. Epub 2014 Nov 12. PMID: 25389056; PMCID: PMC4332815.	1
Dodson RM, Firoozmand A, Hyder O, Tacher V, Cosgrove DP, Bhagat N, Herman JM, Wolfgang CL, Geschwind JF, Kamel IR, Pawlik TM. Impact of sarcopenia on outcomes following intra-arterial therapy of hepatic malignancies. J Gastrointest Surg. 2013 Dec;17(12):2123-32. doi: 10.1007/s11605-013-2348-5. Epub 2013 Sep 25. PMID: 24065364; PMCID: PMC3982291.	1

Sarcopenia in cirrh.Journal of Cachexia, Sarcopenia and Muscleotic patients with and without hepatocellular carcinoma.J. Meza-Junco; A. Montano-Loza; C. Prado; J. Lieffers; V. Baracos; V. Bain; M. Sawyer	1
Dello SA, Lodewick TM, van Dam RM, Reisinger KW, van den Broek MA, von Meyenfeldt MF, Bemelmans MH, Olde Damink SW, Dejong CH. Sarcopenia negatively affects preoperative total functional liver volume in patients undergoing liver resection. HPB (Oxford). 2013 Mar;15(3):165-9. doi: 10.1111/j.1477-2574.2012.00517.x. Epub 2012 Jun 21. PMID: 23020663; PMCID: PMC3572275.	1
Heil J, Heid F, Bechstein WO, Björnsson B, Brismar TB, Carling U, Erdmann J, Fretland ÅA, Grunhagen D, Hana RA, Hohmann J, Linke R, Meyer Y, Nawawi A, Olthof PB, Sandström P, Schnitzbauer AA, Sparrelid E, Verhoef C, Metrakos P, Schadde E. Sarcopenia predicts reduced liver growth and reduced resectability in patients undergoing portal vein embolization before liver resection - A DRAGON collaborative analysis of 306 patients. HPB (Oxford). 2022 Mar;24(3):413-421. doi: 10.1016/j.hpb.2021.08.818. Epub 2021 Aug 16. PMID: 34526229.	1
Sarcopenia predicts survival in patients with advanced hepatocellular carcinoma treated with sorafenib<br>Antonelli, G. et al.Digestive and Liver Disease, Volume 50, Issue 1, 14.DOI:https://doi.org/10.1016/j.dld.2018.01.028	1
Xiao LS, Li RN, Cui H, Hong C, Huang CY, Li QM, Hu CY, Dong ZY, Zhu HB, Liu L. Use of computed tomography-derived body composition to determine the prognosis of patients with primary liver cancer treated with immune checkpoint inhibitors: a retrospective cohort study. BMC Cancer. 2022 Jul 6;22(1):737. doi: 10.1186/s12885-022-09823-7. PMID: 35794525; PMCID: PMC9258103.	1
Loosen SH, Jördens MS, Schoon B, Antoch G, Luedde T, Minko P, Loberg C, Roderburg C. Sarcopenia indicate poor survival in patients undergoing transarterial chemoembolization (TACE) for hepatic malignancies. J Cancer Res Clin Oncol. 2023 Aug;149(9):6181-6190. doi: 10.1007/s00432-022-04519-8. Epub 2023 Jan 23. PMID: 36689060; PMCID: PMC10356883.	1
Baseline Sarcopenia is Associated with Lack of Response to Therapy, Liver Decompensation and High Mortality in Hepatocellular Carcinoma Patients	2
Hashida R, Kawaguchi T, Koya S, Hirota K, Goshima N, Yoshiyama T, Otsuka T, Bekki M, Iwanaga S, Nakano D, Niizeki T, Matsuse H, Kawaguchi A, Shiba N, Torimura T. Impact of cancer rehabilitation on the prognosis of patients with hepatocellular carcinoma. Oncol Lett. 2020 Mar;19(3):2355-2367. doi: 10.3892/ol.2020.11345. Epub 2020 Jan 24. PMID: 32194735; PMCID: PMC7039060.	2
Bettinger D, Spode R, Glaser N, Buettner N, Boettler T, Neumann-Haefelin C, Brunner TB, Gkika E, Maruschke L, Thimme R, Schultheiss M. Survival benefit of transarterial chemoembolization in patients with metastatic hepatocellular carcinoma: a single center experience. BMC Gastroenterol. 2017 Aug 10;17(1):98. doi: 10.1186/s12876-017-0656-z. PMID: 28797231; PMCID: PMC5553671.	2
Loosen SH, Schulze-Hagen M, Bruners P, Tacke F, Trautwein C, Kuhl C, Luedde T, Roderburg C. Sarcopenia Is a Negative Prognostic Factor in Patients Undergoing Transarterial Chemoembolization (TACE) for Hepatic Malignancies. Cancers (Basel). 2019 Oct 8;11(10):1503. doi: 10.3390/cancers11101503. PMID: 31597337; PMCID: PMC6827165.	2
Yabusaki N, Fujii T, Yamada S, Suzuki K, Sugimoto H, Kanda M, Nakayama G, Koike M, Fujiwara M, Kodera Y. Adverse impact of low skeletal muscle index on the prognosis of hepatocellular carcinoma after hepatic resection. Int J Surg. 2016 Jun;30:136-42. doi: 10.1016/j.ijsu.2016.04.049. Epub 2016 May 4. PMID: 27154615.	3

Acosta LF, Galuppo R, García CR, Villacorta E, Dugan A, Castellanos AL, Gedaly R, Lee JT. Association Between Sarcopenia and AFP Level in Patients Undergoing Liver Transplantation for Hepatocellular Carcinoma. J Surg Res. 2019 Jun;238:10-15. doi: 10.1016/j.jss.2019.01.007. Epub 2019 Feb 2. PMID: 30721781.	3
Association of Sarcopenia and Body Composition with Short-term Outcomes after Liver Resection for Malignant Tumors	3
Badran H, Elsabaawy MM, Ragab A, Aly RA, Alsebaey A, Sabry A. Baseline Sarcopenia is Associated with Lack of Response to Therapy, Liver Decompensation and High Mortality in Hepatocellular Carcinoma Patients. Asian Pac J Cancer Prev. 2020 Nov 1;21(11):3285-3290. doi: 10.31557/APJCP.2020.21.11.3285. PMID: 33247686; PMCID: PMC8033124.	3
Clinical impact of the changes of muscle volume during various systemic therapies in patients with unresectable hepatocellular carcinoma.10.1200/JCO.2022.40.16_suppl.e16177 Journal of Clinical Oncology 40, no. 16_suppl (June 01, 2022) e16177-e16177.	3
Hiraoka A, Kumada T, Kariyama K, Tada T, Tani J, Fukunishi S, Atsukawa M, Hirooka M, Tsuji K, Ishikawa T, Takaguchi K, Itobayashi E, Tajiri K, Shimada N, Shibata H, Ochi H, Kawata K, Yasuda S, Toyoda H, Ohama H, Nouso K, Tsutsui A, Nagano T, Itokawa N, Hayama K, Arai T, Imai M, Koizumi Y, Nakamura S, Joko K, Michitaka K, Hiasa Y, Kudo M; Real-life Practice Experts for HCC (RELPEC) Study Group and HCC 48 Group (hepatocellular carcinoma experts from 48 clinics in Japan). Clinical importance of muscle volume in lenvatinib treatment for hepatocellular carcinoma: Analysis adjusted with inverse probability weighting. J Gastroenterol Hepatol. 2021 Jul;36(7):1812-1819. doi: 10.1111/jgh.15336. Epub 2020 Nov 29. PMID: 33171524; PMCID: PMC8359359.	3
Matsumoto H, Tsuchiya K, Nakanishi H, Hayakawa Y, Yasui Y, Uchihara N, Suzuki K, Tanaka Y, Miyamoto H, Ishido S, Yamada M, Keitoku T, Nobusawa T, Higuchi M, Takaura K, Tanaka S, Maeyashiki C, Tamaki N, Takahashi Y, Kurosaki M, Asahina Y, Okamoto R, Izumi N. Clinical Usefulness of Monitoring Muscle Volume during Atezolizumab Plus Bevacizumab Therapy in Patients with Unresectable Hepatocellular Carcinoma. Cancers (Basel). 2022 Jul 21;14(14):3551. doi: 10.3390/cancers14143551. PMID: 35884610; PMCID: PMC9322937.	3
Hirota K, Kawaguchi T, Koya S, Nagamatsu A, Tomita M, Hashida R, Nakano D, Niizeki T, Matsuse H, Shiba N, Torimura T. Clinical utility of the Liver Frailty Index for predicting muscle atrophy in chronic liver disease patients with hepatocellular carcinoma. Hepatol Res. 2020 Mar;50(3):330-341. doi: 10.1111/hepr.13453. Epub 2019 Dec 29. PMID: 31721387.	3
Sugama Y, Miyanishi K, Osuga T, Tanaka S, Hamaguchi K, Ito R, Sakamoto H, Kubo T, Ohnuma H, Murase K, Takada K, Kobune M, Kato J. Combination of psoas muscle mass index and neutrophil/lymphocyte ratio as a prognostic predictor for patients undergoing nonsurgical hepatocellular carcinoma therapy. JGH Open. 2021 Nov 19;5(12):1335-1343. doi: 10.1002/jgh3.12676. PMID: 34950776; PMCID: PMC8674551.	3
Hayashi H, Shimizu A, Kubota K, Notake T, Masuo H, Yoshizawa T, Hosoda K, Sakai H, Yasukawa K, Soejima Y. Combination of sarcopenia and prognostic nutritional index to predict long-term outcomes in patients undergoing initial hepatectomy for hepatocellular carcinoma. Asian J Surg. 2023 Feb;46(2):816-823. doi: 10.1016/j.asjsur.2022.07.122. Epub 2022 Aug 10. PMID: 35961897.	3
Toshida K, Itoh S, Tomiyama T, Morinaga A, Kosai Y, Tomino T, Kurihara T, Nagao Y, Morita K, Harada N, Yoshizumi T. Comparison of the prognostic effect of sarcopenia on atezolizumab plus bevacizumab and lenvatinib therapy in hepatocellular carcinoma patients. JGH Open. 2022 Jun 8;6(7):477-486. doi: 10.1002/jgh3.12777. PMID: 35822124; PMCID: PMC9260215.	3

Seror M, Sartoris R, Hobeika C, Bouattour M, Paradis V, Rautou PE, Soubrane O, Vilgrain V, Cauchy F, Ronot M. Computed Tomography-Derived Liver Surface Nodularity and Sarcopenia as Prognostic Factors in Patients with Resectable Metabolic Syndrome-Related Hepatocellular Carcinoma. Ann Surg Oncol. 2021 Jan;28(1):405-416. doi: 10.1245/s10434-020-09143-9. Epub 2020 Sep 23. PMID: 32965614.	3
Itoh S, Shirabe K, Matsumoto Y, Yoshiya S, Muto J, Harimoto N, Yamashita Y, Ikegami T, Yoshizumi T, Nishie A, Maehara Y. Effect of body composition on outcomes after hepatic resection for hepatocellular carcinoma. Ann Surg Oncol. 2014 Sep;21(9):3063-8. doi: 10.1245/s10434-014-3686-6. Epub 2014 Apr 10. PMID: 24719020.	3
Itoh S, Shirabe K, Matsumoto Y, Yoshiya S, Muto J, Harimoto N, Yamashita Y, Ikegami T, Yoshizumi T, Nishie A, Maehara Y. Effect of body composition on outcomes after hepatic resection for hepatocellular carcinoma. Ann Surg Oncol. 2014 Sep;21(9):3063-8. doi: 10.1245/s10434-014-3686-6. Epub 2014 Apr 10. PMID: 24719020.	3
Itoh S, Yoshizumi T, Kimura K, Okabe H, Harimoto N, Ikegami T, Uchiyama H, Shirabe K, Nishie A, Maehara Y. Effect of Sarcopenic Obesity on Outcomes of Living-Donor Liver Transplantation for Hepatocellular Carcinoma. Anticancer Res. 2016 Jun;36(6):3029-34. PMID: 27272822.	3
Arı D, Dağlı M, Gökcan H, Ökten S, Kaçar S, Bostancı B, Akdoğan M. OP-004. Effects of pretransplant sarcopenia to clinical outcomes after liver transplantation. Turk J Gastroenterol. 2019 Apr;30(supp1):S6. doi: 10.5152/tjg.2019.04. Epub 2019 Apr 1. PMCID: PMC7021037.	3
Dhooge M, Coriat R, Mir O, Perkins G, Brezault C, Boudou-Rouquette P, Goldwasser F, Chaussade S. Feasibility of gemcitabine plus oxaliplatin in advanced hepatocellular carcinoma patients with Child-Pugh B cirrhosis. Oncology. 2013;84(1):32-8. doi: 10.1159/000342763. Epub 2012 Oct 16. PMID: 23076239.	3
Mir O, Coriat R, Boudou-Rouquette P, Ropert S, Durand JP, Cessot A, Mallet V, Sogni P, Chaussade S, Pol S, Goldwasser F. Gemcitabine and oxaliplatin as second-line treatment in patients with hepatocellular carcinoma pre-treated with sorafenib. Med Oncol. 2012 Dec;29(4):2793-9. doi: 10.1007/s12032-012-0208-x. Epub 2012 Mar 17. PMID: 22427209.	3
Kroh A, Uschner D, Lodewick T, Eickhoff RM, Schöning W, Ulmer FT, Neumann UP, Binnebösel M. Impact of body composition on survival and morbidity after liver resection in hepatocellular carcinoma patients. Hepatobiliary Pancreat Dis Int. 2019 Feb;18(1):28-37. doi: 10.1016/j.hbpd.2018.07.008. Epub 2018 Jul 26. PMID: 30115516.	3
Grąt K, Pacho R, Grąt M, Krawczyk M, Zieniewicz K, Rowiński O. Impact of Body Composition on the Risk of Hepatocellular Carcinoma Recurrence After Liver Transplantation. J Clin Med. 2019 Oct 13;8(10):1672. doi: 10.3390/jcm8101672. PMID: 31614892; PMCID: PMC6832484.	3
Endo K, Kuroda H, Kanazawa J, Sato T, Fujiwara Y, Abe T, Sato H, Kooka Y, Oikawa T, Sawara K, Takikawa Y. Impact of Grip Strength in Patients with Unresectable Hepatocellular Carcinoma Treated with Lenvatinib. Cancers (Basel). 2020 Aug 3;12(8):2146. doi: 10.3390/cancers12082146. PMID: 32756366; PMCID: PMC7465794.	3
Bekki T, Abe T, Amano H, Hattori M, Kobayashi T, Nakahara M, Ohdan H, Noriyuki T. Impact of low skeletal muscle mass index and perioperative blood transfusion on the prognosis for HCC following curative resection. BMC Gastroenterol. 2020 Oct 7;20(1):328. doi: 10.1186/s12876-020-01472-z. PMID: 33028209; PMCID: PMC7539410.	3
Beumer BR, van Vugt JLA, Sapisochin G, Yoon P, Bongini M, Lu D, Xu X, De Simone P, Pintore L, Golse N, Nowosad M, Bennet W, Tsochatzis E, Koutli E, Abbassi F, Claasen MPAW, Merli M, O'Rourke J, Gambato M, Benito A, Majumdar A, Tan EK, Ebadi M, Montano-Loza AJ, Berenguer M, Metselaar HJ, Polak WG, Mazzaferro V, IJzermans JNM; Collaborators. Impact of	3

muscle mass on survival of patients with hepatocellular carcinoma after liver transplantation beyond the Milan criteria. J Cachexia Sarcopenia Muscle. 2022 Oct;13(5):2373-2382. doi: 10.1002/jcsm.13053. Epub 2022 Aug 10. PMID: 36622940; PMCID: PMC9530497.	
Hiraoka A, Otsuka Y, Kawasaki H, Izumoto H, Ueki H, Kitahata S, Aibiki T, Okudaira T, Yamago H, Miyamoto Y, Iwasaki R, Tomida H, Mori K, Miyata H, Tsubouchi E, Kishida M, Hirooka M, Abe M, Matsuura B, Ninomiya T, Mori I, Hiasa Y, Michitaka K. Impact of muscle volume and muscle function decline in patients undergoing surgical resection for hepatocellular carcinoma. J Gastroenterol Hepatol. 2018 Jun;33(6):1271-1276. doi: 10.1111/jgh.14058. Epub 2018 Jan 19. PMID: 29193248.	3
Kobayashi A, Kaido T, Hamaguchi Y, Okumura S, Taura K, Hatano E, Okajima H, Uemoto S. Impact of postoperative changes in sarcopenic factors on outcomes after hepatectomy for hepatocellular carcinoma. J Hepatobiliary Pancreat Sci. 2016 Jan;23(1):57-64. doi: 10.1002/jhbp.302. Epub 2015 Dec 3. PMID: 26572789.	3
Takada H, Kurosaki M, Nakanishi H, Takahashi Y, Itakura J, Tsuchiya K, Yasui Y, Tamaki N, Takaura K, Komiyama Y, Higuchi M, Kubota Y, Wang W, Okada M, Enomoto N, Izumi N. Impact of pre-sarcopenia in sorafenib treatment for advanced hepatocellular carcinoma. PLoS One. 2018 Jun 18;13(6):e0198812. doi: 10.1371/journal.pone.0198812. PMID: 29912922; PMCID: PMC6005492.	3
Omiya S, Komatsu S, Kido M, Kuramitsu K, Gon H, Fukushima K, Urade T, So S, Sofue K, Yano Y, Sakai Y, Yanagimoto H, Toyama H, Ajiki T, Fukumoto T. Impact of Sarcopenia as a Prognostic Factor on Reductive Hepatectomy for Advanced Hepatocellular Carcinoma. Anticancer Res. 2021 Nov;41(11):5775-5783. doi: 10.21873/anticanres.15394. PMID: 34732451.	3
Kaido T, Ogawa K, Fujimoto Y, Ogura Y, Hata K, Ito T, Tomiyama K, Yagi S, Mori A, Uemoto S. Impact of sarcopenia on survival in patients undergoing living donor liver transplantation. Am J Transplant. 2013 Jun;13(6):1549-56. doi: 10.1111/ajt.12221. Epub 2013 Apr 19. PMID: 23601159.	3
Kaido T, Hamaguchi Y, Uemoto S. Significance of preoperative sarcopenia to liver surgery. Hepatobiliary Surg Nutr. 2019 Feb;8(1):59-62. doi: 10.21037/hbsn.2018.11.05. PMID: 30881967; PMCID: PMC6383021.	3
Yang J, Chen K, Zheng C, Chen K, Lin J, Meng Q, Chen Z, Deng L, Yu H, Deng T, Bo Z, He Q, Wang Y, Chen G. Impact of sarcopenia on outcomes of patients undergoing liver resection for hepatocellular carcinoma. J Cachexia Sarcopenia Muscle. 2022 Oct;13(5):2383-2392. doi: 10.1002/jcsm.13040. Epub 2022 Jul 19. PMID: 35854105; PMCID: PMC9530540.	3
Salman A, Salman M, Moustafa A, Shaaban HE, El-Mikkawy A, Labib S, Youssef A, Omar MG, Matter M, Elkassar H. Impact of Sarcopenia on Two-Year Mortality in Patients with HCV-Associated Hepatocellular Carcinoma After Radiofrequency Ablation. J Hepatocell Carcinoma. 2021 Apr 29;8:313-320. doi: 10.2147/JHC.S300680. PMID: 33954153; PMCID: PMC8092617.	3
Akce M, Liu Y, Zakka K, Martini DJ, Draper A, Alese OB, Shaib WL, Wu C, Wedd JP, Sellers MT, Bilen MA, El-Rayes BF. Impact of Sarcopenia, BMI, and Inflammatory Biomarkers on Survival in Advanced Hepatocellular Carcinoma Treated With Anti-PD-1 Antibody. Am J Clin Oncol. 2021 Feb 1;44(2):74-81. doi: 10.1097/COC.0000000000000787. PMID: 33350681.	3
Kobayashi A, Kaido T, Hamaguchi Y, Okumura S, Shirai H, Yao S, Kamo N, Yagi S, Taura K, Okajima H, Uemoto S. Impact of Sarcopenic Obesity on Outcomes in Patients Undergoing Hepatectomy for Hepatocellular Carcinoma. Ann Surg. 2019 May;269(5):924-931. doi: 10.1097/SLA.0000000000002555. PMID: 29064889.	3

Kim N, Yu JI, Park HC, Yoo GS, Choi C, Hong JY, Lim HY, Lee J, Choi MS, Lee JE, Kim K. Incorporating sarcopenia and inflammation with radiation therapy in patients with hepatocellular carcinoma treated with nivolumab. Cancer Immunol Immunother. 2021 Jun;70(6):1593-1603. doi: 10.1007/s00262-020-02794-3. Epub 2020 Nov 24. PMID: 33231725.	3
Fujita M, Abe K, Kuroda H, Oikawa T, Ninomiya M, Masamune A, Okumoto K, Katsumi T, Sato W, Iijima K, Endo T, Fukuda S, Tanabe N, Numao H, Takikawa Y, Ueno Y, Ohira H. Influence of skeletal muscle volume loss during lenvatinib treatment on prognosis in unresectable hepatocellular carcinoma: a multicenter study in Tohoku, Japan. Sci Rep. 2022 Apr 20;12(1):6479. doi: 10.1038/s41598-022-10514-3. PMID: 35444161; PMCID: PMC9021276.	3
Alsebaey A, Sabry A, Rashed HS, Elsabaawy MM, Ragab A, Aly RA, Badran H. MELD-Sarcopenia is Better than ALBI and MELD Score in Patients with Hepatocellular Carcinoma Awaiting Liver Transplantation. Asian Pac J Cancer Prev. 2021 Jul 1;22(7):2005-2009. doi: 10.31557/APJCP.2021.22.7.2005. PMID: 34319020; PMCID: PMC8607083.	3
Muscle Steatosis is an Independent Predictor of Postoperative Complications in Patients with Hepatocellular Carcinoma	3
Hiraoka A, Hirooka M, Koizumi Y, Izumoto H, Ueki H, Kaneto M, Kitahata S, Aibiki T, Tomida H, Miyamoto Y, Yamago H, Suga Y, Iwasaki R, Mori K, Miyata H, Tsubouchi E, Kishida M, Ninomiya T, Abe M, Matsuura B, Kawasaki H, Hiasa Y, Michitaka K. Muscle volume loss as a prognostic marker in hepatocellular carcinoma patients treated with sorafenib. Hepatol Res. 2017 May;47(6):558-565. doi: 10.1111/hepr.12780. Epub 2016 Aug 30. PMID: 27480045.	3
Neodadjuvant therapy influences sceletal muscle mass after liver resection for malignancies.T. Bajric; D. Wagner; F. Faschinger; G. Werkgartner; P. Kornprat; P. Schemmer; H. J. Mischinger.European Journal of Surgical Oncology	3
Shiba S, Shibuya K, Katoh H, Koyama Y, Okamoto M, Abe T, Ohno T, Nakano T. No Deterioration in Clinical Outcomes of Carbon Ion Radiotherapy for Sarcopenia Patients with Hepatocellular Carcinoma. Anticancer Res. 2018 Jun;38(6):3579-3586. doi: 10.21873/anticanres.12631. PMID: 29848713.	3
Sano A, Tsuge S, Kakazu E, Iwata T, Ninomiya M, Tsuruoka M, Inoue J, Masamune A. Plasma free amino acids are associated with sarcopenia in the course of hepatocellular carcinoma recurrence. Nutrition. 2021 Apr;84:111007. doi: 10.1016/j.nut.2020.111007. Epub 2020 Sep 2. PMID: 33745507.	3
Pre-operative assessment of muscle mass to predict prognosis in patient with hepatocellular carcinoma.R. Kubota; T. Kumamoto; K. Takeda; F. Asano; Y. Sawada; Y. Ota; Y. Homma; R. Mori; R. Matsuyama; H. Akiyama; I. Endo.	3
Hamaguchi Y, Kaido T, Okumura S, Ito T, Fujimoto Y, Ogawa K, Mori A, Hammad A, Hatano E, Uemoto S. Preoperative intramuscular adipose tissue content is a novel prognostic predictor after hepatectomy for hepatocellular carcinoma. J Hepatobiliary Pancreat Sci. 2015 Jun;22(6):475-85. doi: 10.1002/jhbp.236. Epub 2015 Mar 5. PMID: 25755128.	3
Hamaguchi Y, Kaido T, Okumura S, Ito T, Fujimoto Y, Ogawa K, Mori A, Hammad A, Hatano E, Uemoto S. Preoperative intramuscular adipose tissue content is a novel prognostic predictor after hepatectomy for hepatocellular carcinoma. J Hepatobiliary Pancreat Sci. 2015 Jun;22(6):475-85. doi: 10.1002/jhbp.236. Epub 2015 Mar 5. PMID: 25755128.	3
Preoperative Visceral Adiposity and Muscularity Predict Poor Outcomes after Hepatectomy for Hepatocellular Carcinoma	3
Cheng TY, Lee PC, Chen YT, Chao Y, Hou MC, Huang YH. Pre-sarcopenia determines post-progression outcomes in advanced hepatocellular carcinoma after sorafenib failure. Sci Rep. 2020 Oct 27;10(1):18375. doi: 10.1038/s41598-020-75198-z. PMID: 33110117; PMCID: PMC7591538.	3

Yeh WS, Chiang PL, Kee KM, Chang CD, Lu SN, Chen CH, Wang JH. Pre-sarcopenia is the prognostic factor of overall survival in early-stage hepatoma patients undergoing radiofrequency ablation. Medicine (Baltimore). 2020 Jun 5;99(23):e20455. doi: 10.1097/MD.0000000000020455. PMID: 32501992; PMCID: PMC7306282.	3
Toshima T, Yoshizumi T, Kosai-Fujimoto Y, Inokuchi S, Yoshiya S, Takeishi K, Itoh S, Harada N, Ikegami T, Soejima Y, Mori M. Prognostic Impact of Osteopenia in Patients Who Underwent Living Donor Liver Transplantation for Hepatocellular Carcinoma. World J Surg. 2020 Jan;44(1):258-267. doi: 10.1007/s00268-019-05206-5. PMID: 31624895.	3
Yamasaki T, Saeki I, Yamauchi Y, Matsumoto T, Suehiro Y, Kawaoka T, Uchikawa S, Hiramatsu A, Aikata H, Kobayashi K, Kondo T, Ogasawara S, Chiba T, Takami T, Chayama K, Kato N, Sakaida I. Management of Systemic Therapies and Hepatic Arterial Infusion Chemotherapy in Patients with Advanced Hepatocellular Carcinoma Based on Sarcopenia Assessment. Liver Cancer. 2022 Feb 22;11(4):329-340. doi: 10.1159/000522389. PMID: 35978601; PMCID: PMC9294968.	3
Dong D, Shi JY, Shang X, Liu B, Xu WL, Cui GZ, Wang NY. Prognostic significance of sarcopenia in patients with hepatocellular carcinoma treated with lenvatinib: A retrospective analysis. Medicine (Baltimore). 2022 Feb 4;101(5):e28680. doi: 10.1097/MD.0000000000028680. PMID: 35119010; PMCID: PMC8812594.	3
Nishikawa H, Nishijima N, Enomoto H, Sakamoto A, Nasu A, Komekado H, Nishimura T, Kita R, Kimura T, Iijima H, Nishiguchi S, Osaki Y. Prognostic significance of sarcopenia in patients with hepatocellular carcinoma undergoing sorafenib therapy. Oncol Lett. 2017 Aug;14(2):1637-1647. doi: 10.3892/ol.2017.6287. Epub 2017 May 31. PMID: 28789390; PMCID: PMC5529937.	3
Liao C, Li G, Bai Y, Zhou S, Huang L, Yan M, Qiu F, Chen J, Wang Y, Tian Y, Chen S. Prognostic value and association of sarcopenic obesity and systemic inflammatory indexes in patients with hepatocellular carcinoma following hepatectomy and the establishment of novel predictive nomograms. J Gastrointest Oncol. 2021 Apr;12(2):669-693. doi: 10.21037/jgo-20-341. PMID: 34012658; PMCID: PMC8107609.	3
Imai K, Takai K, Miwa T, Taguchi D, Hanai T, Suetsugu A, Shiraki M, Shimizu M. Rapid Depletions of Subcutaneous Fat Mass and Skeletal Muscle Mass Predict Worse Survival in Patients with Hepatocellular Carcinoma Treated with Sorafenib. Cancers (Basel). 2019 Aug 19;11(8):1206. doi: 10.3390/cancers11081206. PMID: 31430945; PMCID: PMC6721466.	3
Takada H, Amemiya F, Yasumura T, Yoda H, Okuwaki T, Imagawa N, Shimamura N, Tanaka K, Kadokura M, Maekawa S, Enomoto N. Relationship between presarcopenia and event occurrence in patients with primary hepatocellular carcinoma. Sci Rep. 2020 Jun 23;10(1):10186. doi: 10.1038/s41598-020-67147-7. PMID: 32576956; PMCID: PMC7311529.	3
Takagi K, Yagi T, Yoshida R, Shinoura S, Umeda Y, Nobuoka D, Kuise T, Watanabe N, Fujiwara T. Sarcopenia and American Society of Anesthesiologists Physical Status in the Assessment of Outcomes of Hepatocellular Carcinoma Patients Undergoing Hepatectomy. Acta Med Okayama. 2016 Oct;70(5):363-370. doi: 10.18926/AMO/54594. PMID: 27777428.	3
Mardian Y, Yano Y, Ratnasari N, Choridah L, Wasityastuti W, Setyawan NH, Hayashi Y. "Sarcopenia and intramuscular fat deposition are associated with poor survival in Indonesian patients with hepatocellular carcinoma: a retrospective study". BMC Gastroenterol. 2019 Dec 30;19(1):229. doi: 10.1186/s12876-019-1152-4. PMID: 31888500; PMCID: PMC6937974.	3
Chen BB, Liang PC, Shih TT, Liu TH, Shen YC, Lu LC, Lin ZZ, Hsu C, Hsu CH, Cheng AL, Shao YY. Sarcopenia and myosteatosis are associated with survival in patients receiving immunotherapy for advanced hepatocellular carcinoma. Eur Radiol. 2023 Jan;33(1):512-522. doi: 10.1007/s00330-022-08980-4. Epub 2022 Jul 21. PMID: 35864351.	3

Zhao M, Duan X, Han X, Wang J, Han G, Mi L, Shi J, Li N, Yin X, Hou J, Yin F. Sarcopenia and Systemic Inflammation Response Index Predict Response to Systemic Therapy for Hepatocellular Carcinoma and Are Associated With Immune Cells. Front Oncol. 2022 Apr 8;12:854096. doi: 10.3389/fonc.2022.854096. PMID: 35463384; PMCID: PMC9024177.	3
Jang HY, Choi GH, Hwang SH, Jang ES, Kim JW, Ahn JM, Choi Y, Cho JY, Han HS, Lee J, Chung JW, Baeg JY, Jeong SH. Sarcopenia and visceral adiposity predict poor overall survival in hepatocellular carcinoma patients after curative hepatic resection. Transl Cancer Res. 2021 Feb;10(2):854-866. doi: 10.21037/tcr-20-2974. PMID: 35116415; PMCID: PMC8799077.	3
Kim YR, Park S, Han S, Ahn JH, Kim S, Sinn DH, Jeong WK, Ko JS, Gwak MS, Kim GS. Sarcopenia as a predictor of post-transplant tumor recurrence after living donor liver transplantation for hepatocellular carcinoma beyond the Milan criteria. Sci Rep. 2018 May 8;8(1):7157. doi: 10.1038/s41598-018-25628-w. PMID: 29740069; PMCID: PMC5940915.	3
Harimoto N, Shirabe K, Yamashita YI, Ikegami T, Yoshizumi T, Soejima Y, Ikeda T, Maehara Y, Nishie A, Yamanaka T. Sarcopenia as a predictor of prognosis in patients following hepatectomy for hepatocellular carcinoma. Br J Surg. 2013 Oct;100(11):1523-30. doi: 10.1002/bjs.9258. PMID: 24037576.	3
Meza-Junco J, Montano-Loza AJ, Baracos VE, Prado CM, Bain VG, Beaumont C, Esfandiari N, Lieffers JR, Sawyer MB. Sarcopenia as a prognostic index of nutritional status in concurrent cirrhosis and hepatocellular carcinoma. J Clin Gastroenterol. 2013 Nov-Dec;47(10):861-70. doi: 10.1097/MCG.0b013e318293a825. PMID: 23751844.	3
Tan Y, Duan T, Li B, Zhang B, Zhu Y, Yan K, Song J, Lv T, Yang J, Jiang L, Yang J, Wen T, Yan L. Sarcopenia defined by psoas muscle index independently predicts long-term survival after living donor liver transplantation in male recipients. Quant Imaging Med Surg. 2022 Jan;12(1):215-228. doi: 10.21037/qims-21-314. PMID: 34993073; PMCID: PMC8666738.	3
Voron T, Tselikas L, Pietrasz D, Pigneur F, Laurent A, Compagnon P, Salloum C, Luciani A, Azoulay D. Sarcopenia Impacts on Short- and Long-term Results of Hepatectomy for Hepatocellular Carcinoma. Ann Surg. 2015 Jun;261(6):1173-83. doi: 10.1097/SLA.0000000000000743. PMID: 24950264.	3
Imai K, Takai K, Watanabe S, Hanai T, Suetsugu A, Shiraki M, Shimizu M. Sarcopenia Impairs Prognosis of Patients with Hepatocellular Carcinoma: The Role of Liver Functional Reserve and Tumor-Related Factors in Loss of Skeletal Muscle Volume. Nutrients. 2017 Sep 22;9(10):1054. doi: 10.3390/nu9101054. PMID: 28937616; PMCID: PMC5691671.	3
Levolger S, van Vledder MG, Muslem R, Koek M, Niessen WJ, de Man RA, de Bruin RW, Ijzermans JN. Sarcopenia impairs survival in patients with potentially curable hepatocellular carcinoma. J Surg Oncol. 2015 Aug;112(2):208-13. doi: 10.1002/jso.23976. Epub 2015 Aug 12. PMID: 26266324.	3
Sarcopenia in patients undergoing liver transplantation for hepatocellular carcinoma.C. Garcia; L. Acosta; R. Galuppo; E. Villacorta; A. Dugan; A. Castellanos; J. Lee; R. Gedaly.American Journal of Transplantation	3
Salman MA, Omar HSE, Mikhail HMS, Tourky M, El-Ghobary M, Elkassar H, Omar MG, Matter M, Elbasiouny AM, Farag AM, Shaaban HE, Atallah M, Elshabacy O, Salman AA. Sarcopenia increases 1-year mortality after surgical resection of hepatocellular carcinoma. ANZ J Surg. 2020 May;90(5):781-785. doi: 10.1111/ans.15647. Epub 2020 Jan 14. PMID: 31943655.	3
Harimoto N, Yoshizumi T, Shimokawa M, Sakata K, Kimura K, Itoh S, Ikegami T, Ikeda T, Shirabe K, Maehara Y. Sarcopenia is a poor prognostic factor following hepatic resection in patients aged 70 years and older with hepatocellular carcinoma. Hepatol Res. 2016 Nov;46(12):1247-1255. doi: 10.1111/hepr.12674. Epub 2016 Apr 28. PMID: 26880049.	3

Kamachi S, Mizuta T, Otsuka T, Nakashita S, Ide Y, Miyoshi A, Kitahara K, Eguchi Y, Ozaki I, Anzai K. Sarcopenia is a risk factor for the recurrence of hepatocellular carcinoma after curative treatment. Hepatol Res. 2016 Feb;46(2):201-8. doi: 10.1111/hepr.12562. Epub 2015 Aug 18. PMID: 26223826.	3
Antonelli G, Gigante E, Iavarone M, Begini P, Sangiovanni A, Iannicelli E, Biondetti P, Pellicelli AM, Miglioresi L, Marchetti P, Lampertico P, Marignani M. Sarcopenia is associated with reduced survival in patients with advanced hepatocellular carcinoma undergoing sorafenib treatment. United European Gastroenterol J. 2018 Aug;6(7):1039-1048. doi: 10.1177/2050640618781188. Epub 2018 May 31. PMID: 30228892; PMCID: PMC6137588.	3
Sarcopenia negatively impacts long-term outcomes following curative resection for hepatocellular carcinoma: Results of a long-term follow-up study.Cositha Santhakumar, Adam S. Bartlett, Lindsay D. Plank, Cameron I. Wells, Lily Y. Wu, Edward J. Gane, John L. McCall.GastroHep.https://doi.org/10.1002/ygh2.412	3
Mir O, Coriat R, Blanchet B, Durand JP, Boudou-Rouquette P, Michels J, Ropert S, Vidal M, Pol S, Chaussade S, Goldwasser F. Sarcopenia predicts early dose-limiting toxicities and pharmacokinetics of sorafenib in patients with hepatocellular carcinoma. PLoS One. 2012;7(5):e37563. doi: 10.1371/journal.pone.0037563. Epub 2012 May 30. PMID: 22666367; PMCID: PMC3364283.	3
Marasco G, Dajti E, Serenari M, Alemanni LV, Ravaioli F, Ravaioli M, Vestito A, Vara G, Festi D, Golfieri R, Cescon M, Renzulli M, Colecchia A. Sarcopenia Predicts Major Complications after Resection for Primary Hepatocellular Carcinoma in Compensated Cirrhosis. Cancers (Basel). 2022 Apr 12;14(8):1935. doi: 10.3390/cancers14081935. PMID: 35454842; PMCID: PMC9025609.	3
Ha Y, Kim D, Han S, Chon YE, Lee YB, Kim MN, Lee JH, Park H, Rim KS, Hwang SG. Sarcopenia Predicts Prognosis in Patients with Newly Diagnosed Hepatocellular Carcinoma, Independent of Tumor Stage and Liver Function. Cancer Res Treat. 2018 Jul;50(3):843-851. doi: 10.4143/crt.2017.232. Epub 2017 Sep 4. PMID: 28882021; PMCID: PMC6056958.	3
Begini P, Gigante E, Antonelli G, Carbonetti F, Iannicelli E, Anania G, Imperatrice B, Pellicelli AM, Fave GD, Marignani M. Sarcopenia predicts reduced survival in patients with hepatocellular carcinoma at first diagnosis. Ann Hepatol. 2017 Jan-Feb 2017;16(1):107-114. doi: 10.5604/16652681.1226821. PMID: 28051799.	3
Kim H, Choi HZ, Choi JM, Kang BM, Lee JW, Hwang JW. Sarcopenia with systemic inflammation can predict survival in patients with hepatocellular carcinoma undergoing curative resection. J Gastrointest Oncol. 2022 Apr;13(2):744-753. doi: 10.21037/jgo-21-802. PMID: 35557594; PMCID: PMC9086026.	3
Fujiwara N, Nakagawa H, Kudo Y, Tateishi R, Taguri M, Watadani T, Nakagomi R, Kondo M, Nakatsuka T, Minami T, Sato M, Uchino K, Enooku K, Kondo Y, Asaoka Y, Tanaka Y, Ohtomo K, Shiina S, Koike K. Sarcopenia, intramuscular fat deposition, and visceral adiposity independently predict the outcomes of hepatocellular carcinoma. J Hepatol. 2015 Jul;63(1):131-40. doi: 10.1016/j.jhep.2015.02.031. Epub 2015 Feb 24. PMID: 25724366.	3
Yamashima M, Miyaaki H, Honda T, Shibata H, Miuma S, Taura N, Nakao K. Significance of psoas muscle thickness as an indicator of muscle atrophy in patients with hepatocellular carcinoma treated with sorafenib. Mol Clin Oncol. 2017 Sep;7(3):449-453. doi: 10.3892/mco.2017.1321. Epub 2017 Jul 13. PMID: 28781818; PMCID: PMC5530307.	3
Iritani S, Imai K, Takai K, Hanai T, Ideta T, Miyazaki T, Suetsugu A, Shiraki M, Shimizu M, Moriwaki H. Skeletal muscle depletion is an independent prognostic factor for hepatocellular carcinoma. J Gastroenterol. 2015 Mar;50(3):323-32. doi: 10.1007/s00535-014-0964-9. Epub 2014 May 10. PMID: 24817668.	3

Lee J, Cho Y, Park S, Kim JW, Lee IJ. Skeletal Muscle Depletion Predicts the Prognosis of Patients With Hepatocellular Carcinoma Treated With Radiotherapy. Front Oncol. 2019 Oct 15;9:1075. doi: 10.3389/fonc.2019.01075. PMID: 31681607; PMCID: PMC6803501.	3
Imai K, Takai K, Hanai T, Ideta T, Miyazaki T, Kochi T, Suetsugu A, Shiraki M, Shimizu M. Skeletal muscle depletion predicts the prognosis of patients with hepatocellular carcinoma treated with sorafenib. Int J Mol Sci. 2015 Apr 28;16(5):9612-24. doi: 10.3390/ijms16059612. PMID: 25927582; PMCID: PMC4463608.	3
Uojima H, Chuma M, Tanaka Y, Hidaka H, Nakazawa T, Iwabuchi S, Kobayashi S, Hattori N, Ogushi K, Morimoto M, Kagawa T, Tanaka K, Kako M, Koizumi W. Skeletal Muscle Mass Influences Tolerability and Prognosis in Hepatocellular Carcinoma Patients Treated with Lenvatinib. Liver Cancer. 2020 Apr;9(2):193-206. doi: 10.1159/000504604. Epub 2019 Dec 6. Erratum in: Liver Cancer. 2022 Oct 4;11(6):581. PMID: 32399433; PMCID: PMC7206580.	3
Harimoto N, Hoshino H, Muranushi R, Hagiwara K, Yamanaka T, Ishii N, Tsukagoshi M, Igarashi T, Watanabe A, Kubo N, Araki K, Shirabe K. Skeletal Muscle Volume and Intramuscular Adipose Tissue Are Prognostic Predictors of Postoperative Complications After Hepatic Resection. Anticancer Res. 2018 Aug;38(8):4933-4939. doi: 10.21873/anticanres.12810. PMID: 30061272.	3
Nagamatsu A, Kawaguchi T, Hirota K, Koya S, Tomita M, Hashida R, Kida Y, Narao H, Manako Y, Tanaka D, Koga N, Nakano D, Niizeki T, Matsuse H, Torimura T, Shiba N. Slow walking speed overlapped with low handgrip strength in chronic liver disease patients with hepatocellular carcinoma. Hepatol Res. 2019 Dec;49(12):1427-1440. doi: 10.1111/hepr.13405. Epub 2019 Aug 4. PMID: 31273895.	3
Amanuma M, Nagai H, Igarashi Y. Sorafenib Might Induce Sarcopenia in Patients With Hepatocellular Carcinoma by Inhibiting Carnitine Absorption. Anticancer Res. 2020 Jul;40(7):4173-4182. doi: 10.21873/anticanres.14417. PMID: 32620667.	3
Yamaoka K, Kodama K, Kawaoka T, Kosaka M, Johira Y, Shirane Y, Miura R, Yano S, Murakami S, Amioka K, Naruto K, Ando Y, Kosaka Y, Uchikawa S, Uchida T, Fujino H, Nakahara T, Murakami E, Okamoto W, Yamauchi M, Miki D, Imamura M, Takahashi S, Nagao A, Chayama K, Aikata H. The importance of body composition assessment for patients with advanced hepatocellular carcinoma by bioelectrical impedance analysis in lenvatinib treatment. PLoS One. 2022 Jan 18;17(1):e0262675. doi: 10.1371/journal.pone.0262675. PMID: 35041693; PMCID: PMC8765661.	3
Meister FA, Lurje G, Verhoeven S, Wiltberger G, Heij L, Liu WJ, Jiang D, Bruners P, Lang SA, Ulmer TF, Neumann UP, Bednarsch J, Czigany Z. The Role of Sarcopenia and Myosteatosis in Short- and Long-Term Outcomes Following Curative-Intent Surgery for Hepatocellular Carcinoma in a European Cohort. Cancers (Basel). 2022 Jan 30;14(3):720. doi: 10.3390/cancers14030720. PMID: 35158988; PMCID: PMC8833751.	3
Yanagaki M, Haruki K, Taniai T, Igarashi Y, Yasuda J, Furukawa K, Onda S, Shirai Y, Tsunematsu M, Ikegami T. The significance of osteosarcopenia as a predictor of the long-term outcomes in hepatocellular carcinoma after hepatic resection. J Hepatobiliary Pancreat Sci. 2023 Apr;30(4):453-461. doi: 10.1002/jhbp.1246. Epub 2022 Oct 19. PMID: 36181339.	3
Wu CH, Liang PC, Hsu CH, Chang FT, Shao YY, Ting-Fang Shih T. Total skeletal, psoas and rectus abdominis muscle mass as prognostic factors for patients with advanced hepatocellular carcinoma. J Formos Med Assoc. 2021 Jan;120(1 Pt 2):559-566. doi: 10.1016/j.jfma.2020.07.005. Epub 2020 Jul 8. PMID: 32651043.	3

Ebadi M, Moctezuma-Velazquez C, Meza-Junco J, Baracos VE, DunichandHoedl AR, Ghosh S, Sarlieve P, Owen RJ, Kneteman N, Montano-Loza AJ. Visceral Adipose Tissue Radiodensity Is Linked to Prognosis in Hepatocellular Carcinoma Patients Treated with Selective Internal Radiation Therapy. Cancers (Basel). 2020 Feb 4;12(2):356. doi: 10.3390/cancers12020356. PMID: 32033166; PMCID: PMC7072301.	3
Nault JC, Pigneur F, Nelson AC, Costentin C, Tselikas L, Katsahian S, Diao G, Laurent A, Mallat A, Duvoux C, Luciani A, Decaens T. Visceral fat area predicts survival in patients with advanced hepatocellular carcinoma treated with tyrosine kinase inhibitors. Dig Liver Dis. 2015 Oct;47(10):869-76. doi: 10.1016/j.dld.2015.07.001. Epub 2015 Jul 10. PMID: 26211871.	3
Huang YL, Huang MC, Chang CI, Yang LH, Wu CJ, Chiu CC, Chen CY, Hsu JS, Lee KT, Chang WT. Elevated intramuscular adipose tissue content with a high Ishak fibrosis stage (>3) had a negative effect on liver regeneration in cirrhotic patients undergoing portal vein embolization. Kaohsiung J Med Sci. 2023 Feb;39(2):182-190. doi: 10.1002/kjm2.12622. Epub 2022 Nov 17. PMID: 36394149.	3

Table S4. Quality assessment of the included cohort studies using Newcastle-Ottawa Scale [a]

Type of treatment	First author	Selection				Comparability		Outcome			Total points	Overall RoB[b]
		Q1	Q2	Q3	Q4	Q1	Q2	Q1	Q2	Q3		
TACE	Kobayashi 2018	1	1	1	1	1	0	1	0	0	6	High
	Fujita 2019	0	1	1	1	1	0	1	1	0	6	High
	Lim 2021	0	1	1	1	1	1	1	1	0	7	Moderate
	Zhang 2022	0	1	1	1	1	1	1	1	0	7	Moderate
	Yang 2022	0	1	1	1	1	1	1	1	0	7	Moderate
	Chien 2022	0	1	1	1	1	1	1	1	0	7	Moderate
	Roth 2022	1	1	1	1	1	1	1	1	0	8	Moderate
	Li 2023	0	1	1	1	1	1	1	1	0	7	Moderate
	Bannangkoon 2023	0	1	1	1	0	1	1	1	0	6	High
TAE	Lanza 2020	1	1	1	1	1	1	1	1	0	8	Moderate
	Faron 2020	0	1	1	1	1	1	1	1	0	7	Moderate
TARE	Guichet 2021	0	1	1	1	1	1	1	1	0	7	Moderate

RoB, risk of bias.

[a]The judgments of each item in the selection, comparability, and exposure are as follows:

The questions in each domain included:

Selection:

Q1. Representativeness of exposed cohort: 1, truly or somewhat representative of a population-based or multi-center study; 0, selected group of users or lack of description of the derivation of the cohort.

Q2. Selection of non-exposed cohort: 1, drawn from the same community as the exposed cohort; 0, drawn from a different source or lack of description of the derivation of the non-exposed cohort.

Q3. Ascertainment of exposure: 1, secure record or structured interview; 0, written self-report or lack of description of validation.

Q4. Demonstration that outcome of interest was not present at start of study: 1, yes; 0, no.

Comparability:

Q1. Study adjusted for age and sex: 1, yes; 0, no

Q2. study controls for any additional factor: 1, yes; 0, no.

Outcome:

Q1. Assessment of outcome: 1, independent blind assessment, confirmed by medical records or record linkage; 0, self-reported or no description.

Q2. Was follow-up long enough for outcomes to occur: 1, duration of follow-up at least 2.5 years; 0, duration of follow-up < 2.5 years.

Q3. Loss to follow-up rate: 1, complete follow-up of loss to follow-up rate less than 20%; 0, loss to follow-up rate more than 20% or no statement.

[b]We considered studies with a score of 9 stars to be at low RoB, studies that scored 7 or 8 stars at moderate RoB, and those that scored 6 stars or less at high RoB.

Table S5- Subgroup analysis of the prevalence of LSMM in HCC patients treated with transcatheter liver-directed intra-arterial therapies.

Subgroup	N of Records	Patients number	Prevalence (%)	95% confidence interval	I^2(%)
**Overall**	12	2,450	46	38 to 55	94.1
**Treatment regimens**					
TACE	9	2168	43	40 to 45	92.5
TAE	2	200	71	30 to 93	95.9
TARE	1	82	30	22 to 41	0.0
**Study region**					
Asian area	8	1,943	41	33 to 49	91.6
Non-Asian area	4	507	58	35 to 78	95.1
**Population age**					
age ≥ 65	7	1,054	48	33 to 62	95.1
age < 65	5	1,396	45	34 to 56	93.6
**Muscle measured**					
SMI	7	1,643	48	34 to 62	96.6
PMI	3	667	47	43 to 51	0.0
FAMA	2	140	40	23 to 60	81.4

Table S6- Subgroup analysis of HR and 95% confidence interval of overall survival in HCC patients treated with transcatheter liver-directed intra-arterial therapies with and without LSMM

Subgroup	N of Records	Patients number	HR	95% confidence interval	P	I^2(%)	Test for subgroup difference
**Overall**	11	2,388	1.78	1.36 to 2.33	<0.001	75	
**Adjusted**	10	2,107	1.97	1.44 to 2.69	<0.001	78	
**Treatment regimens**							
TACE	8	2,106	1.68	1.23 to 2.30	<0.001	81	P=0.50
TAE	2	200	2.45	1.42 to 4.22	0.001	0	
TARE	1	82	1.94	1.01 to 3.73	0.05	0	
**Study region**							P=0.52
Asian area	7	1,881	1.69	1.19 to 2.40	<0.001	83	
Non-Asian area	4	507	1.97	1.44 to 2.70	<0.001	0	
**Population age**							P=0.57
age ≥65	7	1,054	1.62	1.31 to 2.02	<0.001	0	
age < 65	4	1,334	1.92	1.13 to 3.27	0.02	92	
**Muscle measured**							P=0.24
SMI	6	1,581	1.83	1.20 to 2.80	0.005	84	
PMI	3	667	1.44	1.15 to 1.82	0.001	0	
FFMA	2	140	2.23	1.36 to 3.65	<0.001	0	

Figure S1. Publication bias analysis by using funnel plot for

(A) Prevalence,

(B) HRs of overall survival

(A) Prevalence (Egger's test: p=0.38)

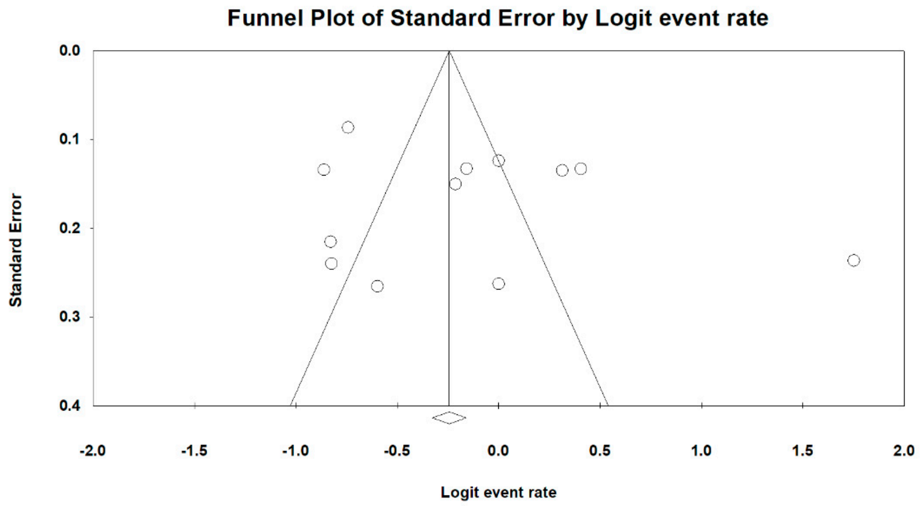

(B) HRs of overall survival (Egger's test: p=0.12)

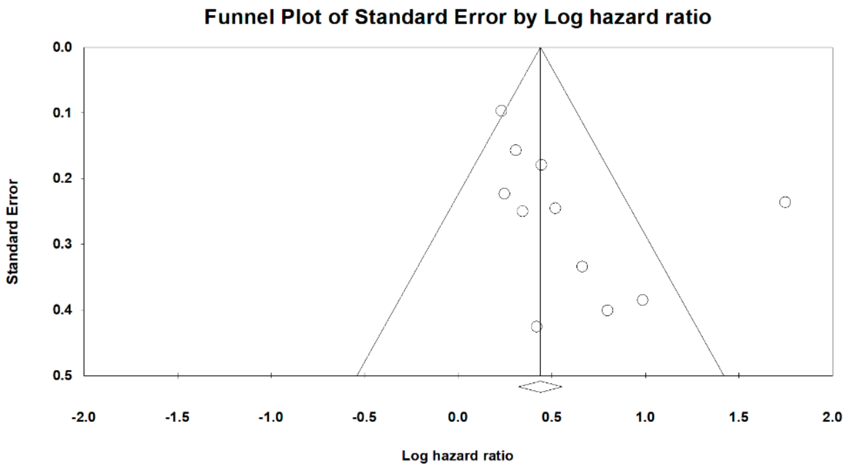

Figure S2. Sensitivity analysis

(A) Prevalence,

(B) Overall survival

(A) Prevalence

Study name	Point	Lower limit	Upper limit
Kobayashi 2018	0.478	0.386	0.571
Fujita 2019	0.464	0.369	0.562
Lanza 2020	0.423	0.353	0.497
Faron 2020	0.460	0.368	0.554
Lim 2021	0.479	0.389	0.572
Guichet 2021	0.477	0.386	0.570
Zhang 2022	0.463	0.367	0.562
Yang 2022	0.472	0.380	0.565
Chien 2022	0.459	0.364	0.558
Roth 2022	0.452	0.361	0.545
Li 2023	0.450	0.361	0.541
Bannangkoon 2023	0.477	0.386	0.570
Pooled	0.463	0.376	0.552

Event rate (95% CI) with study removed

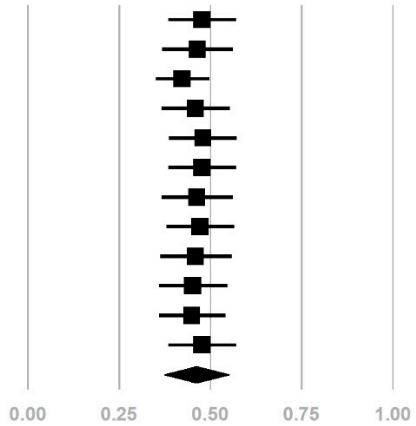

(B) overall survival

Study name	Point	Lower limit	Upper limit
Kobayashi 2018	1.831	1.366	2.455
Fujita 2019	1.854	1.382	2.489
Lim 2021	1.803	1.360	2.392
Zhang 2022	1.821	1.342	2.470
Chien 2022	1.855	1.363	2.524
Lanza 2020	1.758	1.328	2.327
Faron 2020	1.732	1.313	2.285
Guichet 2021	1.773	1.333	2.358
Roth 2022	1.799	1.341	2.413
Li 2023	1.416	1.253	1.599
Bannangkoon 2023	1.879	1.386	2.548
Pooled	1.782	1.363	2.330

Hazard ratio (95% CI) with study removed

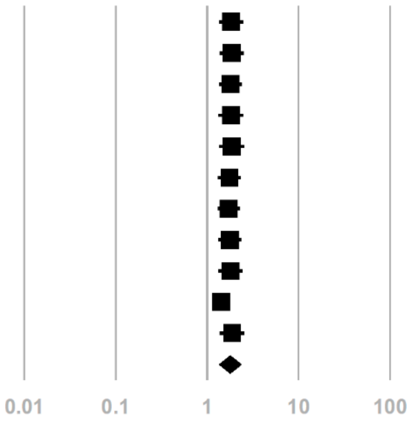

Supplement: Supplementary file 1 [file cancers-16-00319-s001.zip › cancers-2752887-supplementary.pdf]
